# Assessment of Shortcomings of Candidates in the Fellowship of College of Physicians and Surgeons (FCPS)-II (Board/Exit) Examination: A Qualitative Analysis of Dermatology Candidates’ Short and Long Cases With Suggestions for Improvement

**DOI:** 10.7759/cureus.58393

**Published:** 2024-04-16

**Authors:** Bushra Zeeshan, Tariq Rashid

**Affiliations:** 1 Dermatology Unit 1, Allama Iqbal Medical College/Jinnah Hospital, Lahore, PAK; 2 Global Clinical Scholars Research Training, Postgraduate Medical Education, Harvard Medical School, Boston, USA

**Keywords:** board exam, exit exam, clinical exam, dermatology, short case, long case

## Abstract

Background

Clinical examination is the final step for the assessment of a candidate in the Fellowship of College of Physicians and Surgeons-II exam and several stressors are associated with it. After a certain level, the stressors and myths related to this exam increase the chances of poor performance. This study aimed to identify the shortcomings of candidates’ short and long cases with suggestions for improvement.

Methodology

This qualitative research with a thematic analysis approach was conducted at the College of Physicians and Surgeons of Pakistan, Regional Center, Lahore. The data were collected from 20 examiners who assessed 88 candidates’ short and long cases of dermatology through observation and cross-questioning.

Results

The major themes that emerged with more subcategories were poor history taking, poor examination, and poor time management while updated knowledge, rationalization, and enhancement of presentation skills were also highlighted in long cases. The major suggestions to improve the short and long cases were to focus on the command given, maintain the privacy of the patient, and improve training.

Conclusions

This study highlighted many major shortcomings the candidates showed while performing the long and short cases which may facilitate the forthcoming candidates to avoid these weaknesses by following the suggestions for improvements.

## Introduction

Most medical students and even candidates appearing in board exams consider some aspects of their medical education stressful, especially while appearing in the final exit exam which is considered a gateway to their career. The medical exam not only declares a candidate pass or fail but also determines the direction of his/her future and leaves a long-lasting effect on him/her [1].

Clinical examinations are considered stressful not only due to their difficulty but also because of the unpredictability of the type of cases students have to handle, patient’s reactions, and their own performance [2].

In addition to the struggle to meet their own set values, today’s candidates also have to satisfy the demands of their parents and society, which adds to further stress and anxiety [3]. In the domain of education, a high level of anxiety is often experienced by students during performance-related activities such as exams [4,5].

Although some level of anxiety among students is essential to achieve success in exams [6], excessive anxiety can have an adverse effect on their performances [5]. Importantly, in students, a high level of anxiety could have an impact on working memory, reasoning abilities, self-esteem, academic performance, and achievement [7]. Anxiety in students can affect their physical and psychological characteristics causing panic attacks, which make them go blank during exams, nervous, palpation, and could even cause stomach upset [8].

Given the low pass rate of the Fellowship of College of Physicians and Surgeons Pakistan (FCPS)-II exam on the first attempt, this study aims to identify and highlight the shortcomings of candidates appearing in FCPS-II dermatology clinical exam during short and long cases and explore strategies to overcome these shortcomings. The findings may help postgraduate residents appearing in exams and direct them toward success.

## Materials and methods

This study aimed to identify the shortcomings of candidates in short and long cases in FCPS-II dermatology clinical exams and to explore the suggestions for improvement of shortcomings in short and long cases.

A thematic analysis approach was used for this study conducted at the College of Physicians and Surgeons of Pakistan, Regional Center, Lahore, after the end of the FCPS-II clinical dermatology exam. The 88 candidates of the FCPS part-II clinical exam along with 20 examiners were the study sample.

These candidates appearing in this clinical exam as a final step of their examination had already completed their five years of training in dermatology and passed their written exam. These candidates were assessed by 20 examiners of FCPS-II from all over Pakistan. The examiners evaluated candidates using both subjective and objective measures, employing a performance checklist for each task in both short and long cases. After the exam, examiners were provided with questionnaires to address the shortcomings of candidates and offer suggestions for improvement.

In short cases, candidates had 10 minutes to examine an unseen patient according to the given instructions, and after the permission of the examiner, they could proceed to the relevant systematic examination to reach a differential diagnosis. After completing the examination, the candidates briefly described the findings and clinically correlated with the differential diagnosis. Each candidate was cross-questioned by a set of two examiners and evaluated for the four short cases likewise.

In long cases, the candidates had one hour which is usually the most decisive step toward the success of the candidate. In the first 30 minutes, candidates took a detailed history; completed the cutaneous, general physical, and systematic examination; and drew a differential diagnosis. In the next 30 minutes, the candidates had to present a case summary and defend their case with the relevant examination. After the case presentation, the candidates were cross-questioned by a set of two examiners, especially regarding management and recent advances.

## Results

The results revealed that there were some common mistakes the candidates made during the long and short cases. The major themes that emerged with more subcategories were poor history taking and poor examination while updated knowledge, rationalization, and enhancement of presentation skills were also highlighted in long cases. The major suggestions to improve the flaws of the long case were to focus on these weak areas. In short cases, the identified flaws were a lack of reasoning to examine a candidate during a short period. Some overlapping themes that emerged in short cases were poor history-taking skills, handling patients like tools and not as humans, and poor time management. Suggestions were given to improve these target areas.

Candidates in the exam showed several weaknesses in their short cases, as shown in Table 1, the most important among them being the method of clinical approach. Another major issue was the vain attempt to define the lesions without accurately identifying and describing them, where the candidates failed to identify the gross findings. Examination of certain body parts such as axillary lymph nodes, hand web spaces, and trunk sections was usually ignored, not only demonstrating a lack of emphasis but also failure to detect the most important diagnostic features. These drawbacks in the clinical methods not only led to incomplete patient assessments but also inaccurate diagnoses. On the other hand, the candidates showed a dangerous tendency to keep to a narrow-minded approach that even the examiners’ instructions or hints did not broaden their viewpoints. Such a deficiency prevents them from the possibility of brainstorming other diagnoses and changing the tactic to new facts.

**Table 1:** Candidates’ shortcomings in short cases and suggestions for improvement.

Theme	Description	
Poor clinical methods	Indicates that candidates skipped some main elements of identification in short cases	
Subcategories	Description	Statement examples
Poor identification and description of lesions. Skip relevant systematic examination	Candidates missed the main elements of identification	Ignored gross findings. Inability to identify and describe basic lesions. Omission of important relevant areas of examination, i.e., lymph nodes, web spaces in hand examination, and trunk examination
Theme	Description	
Tunnel vision	Contains the fixed diagnostic ideas of the candidates	
Subcategories	Description	Statement examples
Rigid diagnosis. Overlooked the instructions (of the examiners)	Candidates were not willing to change their point of view about the diagnosis given to the patients even after discussion with the examiner	No logical discussion beyond the known diagnosis. Not strictly following the command given
Suggestions for improvements (short cases)		
Theme	Description	
Need for upgraded knowledge	Indicates the need for mock exams for practice	
Subcategories	Description	Statement examples
Training mock exams	Candidates were suggested to attend more training and exposure in outpatient departments (OPDs) in supervision	Supervisors need to spend more time on candidate training in OPDs. Need to have frequent mock exams during training

In light of the referenced drawbacks of the short cases, some primary proposals of solutions were made by the examiners, in pursuance of the positive change, as shown in Table 1. Candidates should first have an essential requirement to sharpen their abilities with practical experience. Besides the regular training sessions, partial practice in handling outpatient department patients will also be beneficial. Second, every candidate is advised to do mock exams often during their training time. These mock tests, on the other hand, serve as a great arena for candidates to practice real situations and diagnose various diseases and the nature of the feedback, which can improve their clinical skills and boost their confidence.

In this exam, the candidates had numerous flaws while performing long cases, as shown in Table 2. Poor history-taking skills which include forgetting to ask about past medical history, gynecological history, Dermatology Life Quality Index, family history, and systematic approaches were some of the key issues that were overlooked. A lack of expertise in scrutinizing important things during history taking was a skill that was lacking. In addition, the examination methods uncovered the basic flaws, i.e., the candidates were poorly skilled in clinical examination and there were situations where they skipped systematic examinations. This flaw caused the inefficiency which, in turn, led to a lack of fluency and methodical ways, demonstrated in the incomplete medical examination of a patient. Moreover, candidates started to behave robot-like and their empathic abilities were weakened with poor bedside manners; hence, they were not able to connect with patients on compassionate grounds. These deficiencies alluded to the fact that the quality of clinical skills as well as the empathetic approach of the candidates in long cases need great improvements.

**Table 2:** Candidates’ shortcomings in long cases and suggestions for improvement.

Flaws in long cases		
Theme	Description	
Poor history taking	Comprises all the deficiencies in taking patient history	
Subcategories	Description	Statement examples
Missing components of history	Candidates lack the skills to cover the major points while taking history	Poor history-taking skills. Missing components of history, especially past medical history, gynecological history, Dermatology Life Quality Index, family history, and relevant systematic inquiry
Theme	Description	
Poor examination skills	Comprises major flaws in examining patients	
Subcategories	Description	Statement examples
Poor clinical skills. Skipping relevant systematic examination	Candidates poorly examined the patients and missed the important components of a flexible examination	Poor examination methods (more acting than fluency, haphazard and not up to the mark. Lack of fluency
Theme	Description	
Lack of empathy	Indicates a lack of compassion toward the patient	
Subcategories	Description	Statement examples
Robotic approach. Poor bedside manners	Candidates did not handle the patients with an empathic approach	A robotic approach to history as if the patient was not a human but only a tool to pass the exam. Very few observed bedside manners
Theme	Description	
Lack of required skills	Includes the professional skills that the candidates lack	
Subcategories	Description	Statement examples
Presentation skills. Time management skills	Most candidates showed poor time management and presentation skills during the clinical exam	The inability to present a comprehensive summary of the case. Took more time in history taking
Theme	Description	
Poor rationalization	Indicates the poor rational abilities of the candidates	
Subcategories	Description	Statement examples
Lack of clinical correlation. Lack of individuation of management	Candidates were unable to give a logical correlation of examination findings with diagnosing logically and practically managing the patient	Clinical correlation of skin lesions was lacking. Poor rationalization regarding patient management (were more theoretical rather than practical)
Theme	Description	
Lack of updated knowledge	Includes the need for updated knowledge	
Subcategories	Description	Statement examples
Unfamiliarity with recent research	Some candidates were not familiar with recent trends in the field of dermatology	Gaps in knowledge and recent advances
Theme	Description	
Fixed and firm ideas	It indicates the inflexible diagnosis of patients made by the candidates	
Subcategories	Description	Statement examples
Obstinate conclusive approach	Candidates were so rigid in giving the diagnosis despite the change in the clinical picture because they had seen the same patient a few days ago in some local tertiary care hospital	Fixed and firm ideas that they had seen the patient before the exam
Suggestions for improvements (long cases)		
Theme	Description	
Updated knowledge	Indicates the need for updated knowledge	
Subcategories	Description	Statement examples
Variety in the exposure of patients. Literature consultation	Candidates were suggested to update their knowledge through different sources	Simulated patients are needed. Reinforce candidates to read the literature for updates on common diseases (for leprosy read more books rather than just Rooks’)
Theme	Description	
Rationalization and uniformity	Includes the importance of logic and sequence in examining a patient	
Subcategories	Description	Statement examples
Logical sequence Individuation in management	Candidates were suggested to rationalize themselves by using standardized procedures	Uniformity of assessment (questions should be same for all candidates). Need to rationalize and individualize the management according to the patient
Theme	Description	
Skill enhancement	Comprises the importance of skill enhancement for a more relevant diagnosis	
Subcategories	Description	Statement examples
Presentation skills. Time management Subsequent training	Candidates were suggested to polish their skills in specific areas	Frequent case presentations during training (detailed and relevant history taking and presentation should be frequently practiced during training since the beginning of the training). History taking should not last for more than 12 minutes (the candidate may ask important remaining questions during the examination)

To deal with the described deficiencies, several suggestions have been offered in Table 2. Initially, the usefulness of up-to-date information is emphasized while dealing with patients of different types and searching for relevant, recent literature. Updated knowledge is a key factor that dermatologists must have to be informed about the current trends and new developments in the dermatology field. Furthermore, rationalization and uniformity are emphasized as the keys to examining patients. Candidates should follow a logical order of investigation and an individualized management system, ensuring the use of protocols to improve their approach. With these, candidates should also work on improving and sharpening their skill set through practice sessions that are specially designed for presentation skills, time management, and shortcomings. Preparation of a 12-minute history taking is recommended as well as the practice of detailed case presentations widely to improve their general clinical performance. By way of these recommendations, candidates will be able to make their way toward an advanced and efficient strategy in long cases, resulting in their expertise in the field of diagnosis and patient management growth.

The major themes of shortcomings in both short and long cases supported by more statements are shown in Table 1 and Table 2, respectively.

## Discussion

In this article, we present the first comprehensive evaluation of the shortcomings observed in candidates by the examiners during the clinical assessment of the FCPS-II exam, which is similar to a board/exit exam. Examination of the candidates during a viva voce or observation is a subjective task and leads to uncertainty and ambiguity. To reduce this stress, candidates should start their preparation with the exam viewpoint which may enable them to focus on the key areas of assessment. Candidates may prepare by completing their syllabus and assessment pattern of the exam by attending different types of training, demonstrations, and case presentations, and, most importantly, by updating their knowledge about the latest advancements in the field.

Although appearing in the final step of the exam after clearing the written test is stressful, candidates need to learn that if they handle patients with care and empathy, then it might be possible that the examiner may overlook knowledge gaps. The results of this study also showed that many candidates mishandled patients, which was noticed by the examiner. They were unable to show compassion toward their patients even though it is a crucial element in doctor-patient relationships [9].

The results of this study also revealed that candidates were unable to take proper medical history, physical examination, and proper diagnosis. To reach a logical diagnosis, all these key areas should be focused upon which usually leads to a systematic examination and appropriate diagnosis [10]. Candidates usually stay up all night the day before the exam which makes them lethargic, drowsy, and inattentive [9]. This inattention may hinder their ability to stay focused and follow the commands of the examiners, especially in short cases.

Results also showed that candidates ignored the important relevant areas of examination, i.e., lymph nodes and web spaces in hand examination. The skin is a complex barrier organ and is constantly exposed to various endogenous and exogenous factors that impact this balanced system, potentially leading to inflammatory skin conditions comprising infections, allergies, or autoimmune diseases [11,12]. It was suggested to the candidates to minutely observe the skin conditions without missing any area in the physical examination.

The findings also showed that candidates were not willing to change their viewpoint about their patients’ diagnoses even after being cross-questioned by the examiner. Such a rigid attitude toward diagnosis compelled the examiner to highlight that the candidates might have seen the patients before the exam. However, it was proved that some of the candidates accidentally had seen some patients in the local tertiary care hospital and they gave an inflexible diagnosis to those patients. If candidates show flexibility in their diagnostic point of view, it may avoid confusion and misdiagnosis which is usually the result of firm adherence to unreasonable rigid criteria.

Ignoring the given commands may affect the assessment of the candidates and show their rigidity, as was highlighted by the examiners in both long and short cases that the candidates used tunnel vision and were very rigid in their diagnosis.

Candidates also failed to defend the differential diagnosis they made during the exam which was an indication of knowledge gaps; therefore, the examiners suggested updating themselves through literature and simulated patients. It was also suggested that candidates attend more training in clinical dermatology than cosmetology workshops to improve their clinical diagnostic skills. It is supported by the literature that training improves the experience and diagnostic skills of a dermatologist [13].

Thus, this study highlighted many major flaws the candidates showed during both long and short cases which may facilitate the forthcoming candidates to avoid these weaknesses by following the suggested suggestions for improvements.

Study limitations

The study limitations include subjective appearance bias by the examiners, single-center data, and lack of sample diversity. Due to the limited existing literature on this topic, critical and constructive comparisons could not be made. Additionally, the absence of longitudinal data and consideration of confounders such as exam stress is another limitation. Future research should employ larger, diverse samples and standardized measurement tools for more reliable findings.

## Conclusions

This study provides valuable insights into the shortcomings that candidates demonstrated during the FCPS-II dermatology clinical exam, which is an exit exam in dermatology. The study findings highlight the critical importance of thorough and timely preparation and a holistic approach for candidates undergoing viva voce or observational assessments in the medical field. Addressing these shortcomings and implementing the suggested improvements will undoubtedly contribute to more effective and comprehensive preparation by the forthcoming candidates. Thus, this approach will not only help the candidates pass the exam but will also make future consultants more proficient, accurate, and empathetic in clinical assessments of their patients, ultimately benefiting patient care and the overall quality of medical practice. However, it is important to acknowledge the study’s limitations, thus underscoring the need for future research with broader and more diverse samples.
